# Accelerators to reduce violence, HIV risk, and early pregnancy among adolescents and young people in Namibia: A cross-sectional analysis of the Violence Against Children & Youth Survey

**DOI:** 10.1371/journal.pgph.0004633

**Published:** 2025-05-20

**Authors:** Madison T. Little, Lucas Hertzog, William E. Rudgard, Elona Toska, Boladé Banougnin, Rachel Yates, David Chipanta, Francis B. Annor, Laura Chiang, Lucie Cluver

**Affiliations:** 1 Centre for Evidence-Based Intervention, Department of Social Policy & Intervention, University of Oxford, Oxford, United Kingdom; 2 Green Templeton College, University of Oxford, Oxford, United Kingdom; 3 Curtin School of Population Health, Curtin University, Perth, Australia; 4 Centre for Social Science Research, University of Cape Town, Cape Town, South Africa; 5 UNFPA West & Central African Regional Office, Dakar, Senegal; 6 UNAIDS Namibia, Windhoek, Namibia; 7 Division of Violence Prevention, National Center for Injury Prevention & Control, United States of America Centers for Disease Control & Prevention, Atlanta, Georgia, United States of America; 8 Department of Psychiatry & Mental Health, University of Cape Town, Cape Town, South Africa; PLOS: Public Library of Science, UNITED STATES OF AMERICA

## Abstract

Our study applied the INSPIRE Framework – the WHO’s 2016 technical package of evidence-based interventions for addressing violence against children – to identify accelerators for youth in Namibia. Accelerators are protective factors that contribute toward achieving multiple SDG targets. Using nationally representative data from the 2019 Namibia Violence Against Children & Youth Survey (n = 5167), three hypothesised accelerators (food security, parental support, and gender-equitable attitudes) were investigated for their impact on 12 adolescent outcomes. Associations between the hypothesised accelerators and outcomes were assessed using multivariable logistic regressions, and adjusted probabilities, differences, and ratios. Among girls, food security, gender-equitable attitudes, and parental support were accelerators, being associated with lower odds for 8, 6, and 2 outcomes, respectively. When all three were present, the combination was significantly associated with 10 out of 12 outcomes, including >75% lower prevalences of child marriage; > 50% lower prevalences of child abuse, sexual violence victimisation, early sexual debut/early pregnancy, and peer violence victimisation; and >25% lower prevalences of intimate partner violence (IPV) victimisation, not being in school or paid work, mental health distress, inconsistent condom use, and age-disparate or transactional sex. Among boys, gender-equitable attitudes was an accelerator and was significantly associated with 7 out of 10 outcomes, including approximately 50% lower prevalences of sexual violence victimisation, child abuse, age-disparate or transactional sex, IPV victimisation, multiple sexual partners, peer violence victimisation, and inconsistent condom use. Adolescents (especially girls) with access to INSPIRE provisions experience lower rates of violence and HIV-related risks. Implementing interventions on these priority protective factors could accelerate progress in achieving the SDGs for adolescents and young people in Namibia.

## Introduction

For adolescents in Namibia and across the African continent, violence, HIV, and early pregnancy threaten development and longevity. Violence against children and adolescents in Namibia is prevalent in multiple forms, including physical violence (33% of girls, 41% of boys in 2019), emotional violence (11% of girls, 8% of boys), and sexual violence (12% of girls, 7% of boys) [1]. As of 2017, AIDS remained the leading cause of death for Namibian children and adolescents [2] and in 2019, nearly one-in-five adolescent girls experienced early pregnancy and motherhood [3].

Adolescents in Namibia also face a range of deprivations that further threaten their safe transition to adulthood. Rates of both orphanhood and food insecurity significantly increased during the COVID-19 pandemic as well as rates of gender-based violence and early pregnancy [3–6]. In response to the pandemic, innovative interventions are necessary to regain progress to reach key targets in the Sustainable Development Goals (SDGs).

One possible innovation is the application of the INSPIRE Framework, which provides seven strategies to address violence against children and its risk factors [7]. These interventions may be highly effective in reducing violence by addressing multiple pathways of vulnerability. These prevention pathways include establishing and upholding legal protections for children, challenging harmful social norms, fostering safe environments, providing parent/caregiver support, disbursing financial support, and teaching life skills, as well as a response strategy providing psychosocial support to children who experienced violence [7]. Despite the promise of INSPIRE interventions, there has been limited evidence testing their effectiveness in Namibia. Although previous evaluations in the country have focused on poverty reduction through national cash transfer programmes [8–10], there is no evidence on whether social protection, including cash transfers, is associated with violence prevention in this context. It is also unknown whether other INSPIRE interventions, such as parenting interventions or gender-transformative programming, also have violence prevention impacts since no analyses are available from Namibia.

Recent evidence from other countries in the region has also shown that INSPIRE interventions can have additional impacts on several domains of an adolescent’s life, such as health and education [11]. These programmes can be classified as accelerators, which are interventions or provisions (i.e., modifiable protective factors) that contribute toward multiple improved outcomes [12,13]. Together, these accelerators can have cumulative effects, specifically additional intervention impact from being delivered in combination [11]. In Namibia, there is no evidence on whether INSPIRE provisions may qualify as accelerators in further improving violence prevention and other outcomes for adolescents.

To address this gap in the evidence base, this paper aimed to answer two questions: 1) Which INSPIRE provisions (hypothesised accelerators) are associated with lower prevalences of multiple adverse outcomes for adolescent girls and young women and for adolescent boys and young men in Namibia? 2) What are the cumulative effects of the hypothesised accelerators across multiple outcomes?

## Materials & methods

### Study design, setting, & participants

This secondary analysis utilised cross-sectional survey data from the publicly available and nationally representative Namibia 2019 Violence Against Children & Youth Survey (VACS). This survey was administered from March to June 2019 to adolescents and young people aged 13–24 (adolescents aged 13–17 and young people aged 18–24) living in non-institutionalised settings [1]. The survey aims to contribute to evidence-based programming to end violence against women and children (Sustainable Development Goals 5.1-5.2 and 16.2, respectively) [1].

Several studies have been published detailing an overview of the survey and its sampling design, methodology, and statistical procedures [14,15]. VACS data utilise a three-stage, geographically clustered sampling strategy in its random selection of communities, households, and individuals [14]. The sample frame in Namibia was based on data from the 2010 census and the Intercensal Demographic Survey in 2016 [16]. Within selected households, only one individual was included in the survey (randomly selected) and was assigned a sex-specific primary sampling unit [16]. The survey procedures required that the individual speak one of six local languages or English and not have a disability impacting the understanding of, or responding to, the questionnaires [16]. In total, 5191 adolescents and young people aged 13–24 completed the survey. Twenty-four cases (<0.5% of the total sample) were dropped from the dataset due to no linked household survey. The final sample for this paper’s analysis (n = 5167) included 4189 females and 978 males. Overall, the response rate was 88.5% for females and 84.2% for males. The weighted sample represents a population of 514,251 adolescents and young people in Namibia.

The survey also oversampled females living in priority districts (Khomas, Oshikoto, and Zambezi) for PEPFAR’s DREAMS Initiative. This effort is a set of packaged interventions from the US HIV/AIDS bilateral agency to address the multiple, complex pathways that increase HIV risk for adolescent girls and young women [17]. Oversampling is adjusted for in the sample weights and our analysis included an additional variable to account for oversampling [16].

### Variables & measurements

VACS data use validated survey tools that are culturally adapted to each country and piloted in field testing prior to full-scale implementation [18]. This includes adjustments to wording of the questions/answers as well as the addition of questions or modules based on country priorities and context and consultation with key, local stakeholders. Thus, the hypothesised accelerators, outcomes, and covariates are based on scales that have been tested and validated elsewhere. Survey questions and combination strategies for each variable are available in S1 Table.

For this dataset, three hypothesised accelerators (protective factors) were identified that align with three core strategies in the INSPIRE framework: Norms & values (having gender-equitable attitudes), Parent & caregiver support (having parental support), and Income & economic strengthening (having household food security). Adolescents and young people having gender-equitable attitudes was measured as their rejection of social norms on domestic violence and violence against women, more broadly. Parental support was measured as having both good communication and a good relationship with at least one parent they had seen in the previous year. Food security was measured as food being both affordable (adolescent-reported) and sufficient (household-head-reported, not skipping meals or cutting portion sizes). All three hypothesised accelerators had components measured in the past year. Questionnaires and codebooks are publicly available online [19].

Twelve self-reported outcomes were investigated: Intimate partner violence (IPV) victimisation (physical or emotional), peer violence victimisation (physical or emotional), sexual violence victimisation, child abuse victimisation (physical or emotional), multiple sexual partners, inconsistent condom use, age-disparate or transactional sex, early sexual debut (before age 16) or early pregnancy (before age 20), heavy drinking (≥4 alcoholic drinks on one occasion), moderate or severe mental health distress, not being in school or paid work, and child marriage (before age 18). All outcomes were based on self-report with a reference period of the previous year except for heavy drinking and mental distress, which used a reference period of the past 30 days, and child marriage, which used a lifetime reference period due to its low prevalence. These outcomes span four SDGs: 3) Good health & wellbeing; 5) Gender equality; 10) Reduced inequalities; and 16) Peace, justice, & strong institutions.

Covariates included age group (13-17 and 18-24), orphanhood status (having lost one or both parents), urban/rural, female-headed household, and household poverty (lowest two quintiles of the wealth index). Household poverty was computed using principal component analysis and was measured using a scale based on questions related to living standards, which included having improved cooking fuel, private sanitation, safe drinking water, electricity, improved household building material, and household-item ownership.

### Statistical analysis

Analysis was done in Stata 17 and sample code for the analytic approach is available open access [20]. All hypothesised accelerators, outcomes, and covariates were measured as binary indicators, and weighted prevalences, 95% confidence intervals, and sample sizes were presented and stratified by sex; sex differences were evaluated using chi-squared tests. We used Spearman’s correlation to investigate correlations between outcome-pairs. The outcome-pair correlations (S2 Table) show a low correlation (having |r| < 0.30) among >95% of the outcomes; no further adjustments to regressions were required as there are minimal gains of accounting for correlated outcomes when |r| < 0.6 [21].

A series of statistical models were used to ensure the robustness of our findings, including tests to ensure our findings were not due to chance. Univariate logistic regressions were run prior to multivariable logistic regressions to assess the unadjusted associations between the hypothesised accelerators and each outcome separately. Subsequently, complete multivariable logistic regressions were run for each outcome and included all three hypothesised accelerators. All analyses were stratified by gender and all analyses accounted for the three-stage sampling procedure and over-sampling in key districts. The analyses also controlled for sociodemographic covariates and adjusted for multiple hypothesis testing using Westfall-Young stepdown adjusted p-values. Given the number of different protective factors and outcomes being tested, we used the Westfall-Young procedure to improve the confidence that the results were not due to chance, which in turn makes the findings more reliable. Westfall-Young stepdown procedure was chosen for its ability to accommodate complexities such as multiple treatments, correlated outcomes, and different controls between regressions (relevant to measures specific to younger adolescents and offering a balanced control of Type I and II errors) [22–25].

Protective factors were considered as ‘accelerators’ if they were associated with two or more outcomes after correcting for multiple tests [12,13]. Different scenarios were modelled to evaluate how protective factors may be associated with the outcomes. Adjusted outcome probabilities were calculated based on three scenarios: (i) Having no accelerators, (ii) only one significant accelerator, or (iii) all significant accelerators. We also estimated the adjusted prevalence difference and ratio of outcomes contrasting scenarios (ii) and (iii) to the baseline scenario of (i). Sensitivity analyses were conducted by re-running the multivariable logistic regression models including only those variables (hypothesised accelerators and covariates) that were significant to p < 0.05 in the univariate or complete multivariable logistic regressions.

Ethics approval was received for this secondary data analysis by the University of Oxford (SPI_DREC_20–21_050). All participants provided informed consent or assent and for those under age 18, consent from the caregiver was also required. Additional details on the ethics protocol as part of the primary data collection process are specified elsewhere [14,15].

## Results

### Descriptive and outcome data

Sample characteristics (weighted, sex-disaggregated prevalences of hypothesised accelerators, outcomes, and sociodemographic covariates) are provided in Table 1. The mean age for females and males separately was 18 years old with a standard deviation of 3.4 years. Females were significantly more likely to be food secure than their male counterparts (39% vs 28%, p = 0.026), though the vast majority of females and males were food insecure. Males were more likely to have parental support as compared with females (26% vs 18%, p = 0.003). There was no evidence that rates of gender-equitable attitudes differed between females and males (48% vs 40%, p = 0.075).

**Table 1:** Sample characteristics among adolescents and young people aged 13-24 years (includes sample weights).

	Females			Males			Sex Difference
	Weighted %	95% CI	n (sample)	Weighted %	95% CI	n (sample)	*p*-value
**Hypothesised Accelerators**							
Household food security	39	(34, 44)	1464 (4189)	28	(23, 34)	318 (978)	0.026
Parental support	18	(15, 20)	711 (4187)	26	(21, 32)	223 (978)	0.003
Gender-equitable attitudes	48	(42, 54)	2169 (4139)	40	(35, 45)	395 (964)	0.075
							
**Outcomes**							
IPV victimisation (physical or emotional, 12mon)	12	(10, 15)	455 (4189)	12	(9, 16)	137 (978)	0.868
Peer violence victimisation (physical or emotional, 12mon)	23	(19, 26)	730 (4189)	27	(23, 32)	256 (978)	0.095
Sexual violence victimisation (12mon)	11	(9, 13)	386 (4189)	7	(5, 10)	70 (978)	0.031
Child abuse (physical or emotional, 12mon)	23	(20, 26)	869 (4189)	21	(17, 25)	202 (978)	0.451
Multiple sexual partners (12mon)	4	(3, 6)	139 (4114)	15	(12, 18)	139 (950)	<0.001
Inconsistent condom use (12mon)	25	(22, 28)	1182 (4104)	14	(11, 18)	148 (946)	<0.001
Age-disparate or transactional sex (12mon)	14	(12, 17)	700 (4189)	7	(5, 10)	85 (978)	<0.001
Early sex (<16) or early pregnancy (<20) (12mon)	9	(7, 12)	241 (2518)	Small sample sizes preclude reliable estimations.			
Heavy drinking (30 days)	12	(10, 15)	463 (4089)	20	(16, 25)	190 (970)	<0.001
Mental health distress (30 days)	40	(35, 45)	1694 (4178)	22	(17, 27)	236 (978)	<0.001
Not in school or paid work (12mon)	20	(18, 24)	907 (4115)	14	(11, 17)	142 (970)	0.002
Child marriage (before age 18, lifetime)	1	(1,2)	83 (4132)	Small sample sizes preclude reliable estimations.			
							
**Sociodemographic Covariates**							
Age 13–17 (Binary)	42	(39, 46)	1770 (4189)	42	(38, 45)	414 (978)	0.828
Orphanhood	30	(27, 33)	1308 (4186)	27	(23, 30)	277 (978)	0.185
Urban	53	(43, 63)	2206 (4189)	40	(28, 53)	482 (978)	0.230
Household poverty (lowest two quintiles)	38	(29, 47)	1638 (4189)	42	(34, 51)	377 (978)	0.551
Oversampling in priority district	36	(29, 44)	3594 (4189)	28	(18, 42)	317 (978)	0.407
Female-headed household	74	(71, 77)	3039 (4189)	54	(49, 59)	499 (978)	<0.001

CI = Confidence Interval.

Among outcomes, females were more likely to experience sexual violence (11% vs. 7%, p = 0.031), inconsistent condom use (25% vs. 14%, p < 0.001), age-disparate or transactional sex (14% vs. 7%, p < 0.001), mental health distress (40% vs. 22%, p < 0.001), and not being in school or paid work (20% vs. 14%, p = 0.002). In contrast, males were more likely to have multiple sexual partners (15% vs. 4%, p < 0.001) and heavy drinking (20% vs. 12%, p < 0.001). There was no evidence that rates of other outcomes differed between genders. Among covariates, females were more likely to live in a female-headed household (74% vs 54%, p < 0.001) and more than a quarter of the total sample of females and males had experienced the death of at least one parent.

### Multivariable associations among females

The presence of multiple protective factors was associated with lower prevalences of adverse outcomes. Among females, food security, gender-equitable attitudes, and parental support were associated with lower odds for multiple negative outcomes, as shown in multivariable logistic regressions in Table 2. Food security was associated with statistically-significant lower odds for eight adverse outcomes: Physical or emotional intimate partner violence (IPV) victimisation (Adjusted Odds Ratio, AOR = 0.63 (95% Confidence Interval 0.48, 0.85)), sexual violence victimisation (AOR = 0.57 (0.40, 0.83)), physical or emotional child abuse victimisation (AOR = 0.67 (0.50, 0.89)), inconsistent condom use (AOR = 0.67 (0.48, 0.95)), age-disparate or transactional sex (AOR = 0.66 (0.46, 0.93)), mental health distress (AOR = 0.65 (0.49, 0.84)), not in school or paid work (AOR = 0.51 (0.36, 0.73)), and child marriage (AOR = 0.20 (0.08, 0.47)).

**Table 2:** Associations between hypothesised accelerators and outcomes disaggregated by sex (includes all hypothesised accelerators, covariates, and sample weights).

	Females			Males		
	AOR	(95% CI)	p-value	AOR	(95% CI)	p-value
**IPV (physical or emotional; n = 4134F, 964M)**						
Household food security	0.63	(0.48, 0.85)	<0.001	1.05	(0.69, 1.60)	1.00
Parental support	0.91	(0.58, 1.43)	0.96	0.72	(0.33, 1.55)	0.72
Gender-equitable attitudes	0.76	(0.49, 1.16)	0.26	0.44	(0.27, 0.72)	<0.001
**Peer violence (physical or emotional; n = 4134F, 964M)**						
Household food security	0.68	(0.44, 1.03)	0.07	0.51	(0.31, 0.85)	<0.001
Parental support	0.81	(0.52, 1.26)	0.77	0.79	(0.47, 1.33)	0.73
Gender-equitable attitudes	0.58	(0.39, 0.87)	0.01	0.39	(0.24, 0.62)	<0.001
**Sexual violence (n = 4134F, 964M)**						
Household food security	0.57	(0.40, 0.83)	<0.001	0.91	(0.42, 2.01)	1.00
Parental support	1.15	(0.65, 2.05)	0.96	0.56	(0.26, 1.22)	0.28
Gender-equitable attitudes	0.64	(0.47, 0.86)	<0.001	0.39	(0.19, 0.79)	<0.001
**Child abuse (physical or emotional; n = 4134F, 964M)**						
Household food security	0.67	(0.50, 0.89)	0.01	0.72	(0.46, 1.13)	0.53
Parental support	0.55	(0.35, 0.86)	0.01	0.90	(0.51, 1.59)	0.98
Gender-equitable attitudes	0.68	(0.49, 0.93)	0.01	0.34	(0.18, 0.61)	<0.001
**Multiple sexual partners (n = 4067F, 937M)**						
Household food security	0.76	(0.45, 1.29)	0.21	1.04	(0.60, 1.80)	1.00
Parental support	0.68	(0.32, 1.46)	0.72	0.93	(0.52, 1.66)	0.98
Gender-equitable attitudes	1.01	(0.49, 2.08)	0.97	0.43	(0.28, 0.67)	<0.001
**Inconsistent condom use (n = 4057F, 933M)**						
Household food security	0.67	(0.48, 0.95)	0.03	0.93	(0.50, 1.74)	1.00
Parental support	0.75	(0.54, 1.04)	0.29	0.84	(0.42, 1.70)	0.97
Gender-equitable attitudes	1.14	(0.88, 1.47)	0.65	0.47	(0.29, 0.78)	0.01
**Age-disparate or transactional sex (n = 4134F, 964M)**						
Household food security	0.66	(0.46, 0.93)	0.02	1.07	(0.49, 2.34)	1.00
Parental support	0.91	(0.64, 1.29)	0.96	1.81	(0.86, 3.83)	0.17
Gender-equitable attitudes	1.25	(0.84, 1.84)	0.45	0.43	(0.21, 0.88)	0.01
**Early sex (<16) or early pregnancy (<20) (n = 2481F, -)**						
Household food security	0.52	(0.23, 1.19)	0.13	–	–	
Parental support	1.33	(0.59, 2.99)	0.85	–	–	
Gender-equitable attitudes	0.63	(0.41, 0.98)	0.03	–	–	
**Heavy drinking (n = 4034F, 956M)**						
Household food security	1.32	(0.92, 1.89)	0.13	1.04	(0.62, 1.76)	1.00
Parental support	0.71	(0.50, 1.00)	0.12	0.92	(0.57, 1.48)	0.98
Gender-equitable attitudes	1.06	(0.69, 1.62)	0.91	0.68	(0.45, 1.01)	0.04
**Mental distress (n = 4126F, 964M)**						
Household food security	0.65	(0.49, 0.84)	<0.001	1.12	(0.64, 1.95)	0.99
Parental support	0.97	(0.70, 1.37)	0.96	0.68	(0.34, 1.36)	0.46
Gender-equitable attitudes	0.68	(0.49, 0.94)	0.01	0.71	(0.47, 1.06)	0.04
**Not in school or paid work (n = 4064F, 958M)**						
Household food security	0.51	(0.36, 0.73)	<0.001	0.82	(0.53, 1.28)	0.87
Parental support	1.49	(1.00, 2.22)	0.12	1.13	(0.61, 2.06)	0.98
Gender-equitable attitudes	0.90	(0.66, 1.24)	0.75	0.73	(0.44, 1.20)	0.08
**Child marriage (before age 18; n = 4107F, -)**						
Household food security	0.20	(0.08, 0.47)	<0.001	–	–	
Parental support	0.27	(0.10, 0.77)	0.02	–	–	
Gender-equitable attitudes	0.27	(0.11, 0.67)	0.01	–	–	

AOR = Adjusted Odds Ratio; CI = Confidence Interval; Wyoung = Westfall-Young adjusted p-value for multiple hypothesis testing; IPV = Intimate Partner Violence; F = Female Sample; M = Male Sample.

Parental support was associated with lower odds for two adverse outcomes: Physical or emotional child abuse (AOR = 0.55 (0.35, 0.86)) and child marriage (AOR = 0.27 (0.10, 0.77)). Lastly, gender-equitable attitudes were associated with lower odds for six adverse outcomes: Physical or emotional peer violence victimisation (AOR = 0.58 (0.38, 0.87)), sexual violence victimisation (AOR = 0.64 (0.47, 0.86)), physical or emotional child abuse victimisation (AOR = 0.68 (0.48, 0.93)), early sexual debut or early pregnancy (AOR = 0.63 (0.41, 0.98)), mental health distress (AOR = 0.68 (0.49, 0.94)), and child marriage (AOR = 0.27 (0.11, 0.67)). In total, household food security, parental support, and gender-equitable attitudes were associated with lower odds for 10 out of the 12 negative outcomes. There was no statistically-significant association between the hypothesised accelerators and having multiple sexual partners or heavy drinking.

### Multivariable logistic associations among males

Among males (see Table 2), household food security was associated with statistically-significant lower odds for peer violence victimisation (Adjusted Odds Ratio, AOR = 0.51 (95% Confidence Interval 0.31, 0.85)). Parental support was not associated with any outcomes among males. Gender-equitable attitudes was associated with statistically-significant lower odds for 7 out of 10 adverse outcomes: Physical or emotional IPV victimisation (AOR = 0.44 (0.27, 0.72)), physical or emotional peer violence victimisation (AOR = 0.39 (0.24, 0.62)), sexual violence victimisation (AOR = 0.39 (0.19, 0.79)), physical or emotional child abuse (AOR = 0.34 (0.18, 0.61)), multiple sexual partners (AOR = 0.43 (0.28, 0.67)), inconsistent condom use (AOR = 0.47 (0.29, 0.78)), and age-disparate or transactional sex (AOR = 0.43 (0.21, 0.88)).

### Adjusted probabilities from accelerators

**Fig 1:**
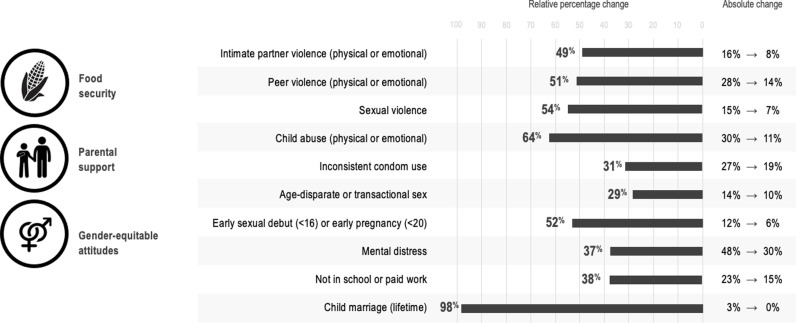
Cumulative associations of experiencing negative outcomes in the presence of no or all accelerators among females.

**Fig 2:**
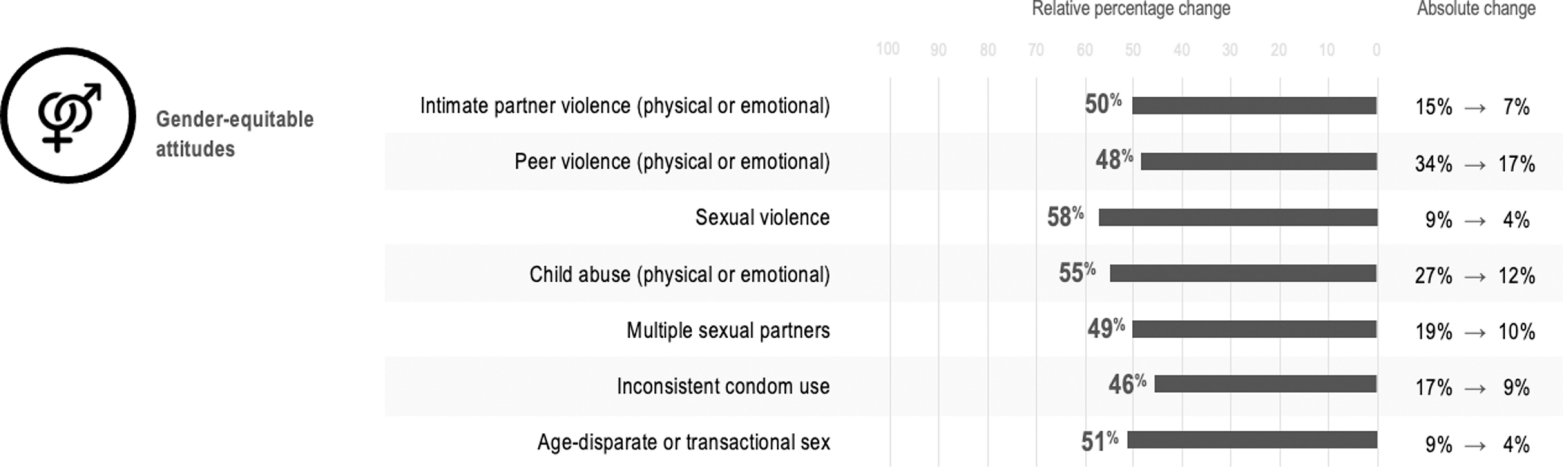
Cumulative associations of experiencing negative outcomes in the presence of gender-equitable attitudes among males.

Tables 3–4 provides sex-specific probability differences (adjusted probabilities and adjusted prevalence differences and ratios). These tables show adjusted probabilities for outcomes based on the presence of (i) no accelerators, (ii) each accelerator individually, and (iii) all accelerators together (three for females, one for males). As an example among females, the estimated prevalence of child abuse was 30% in the absence of accelerators, 23% with food security alone, 20% with parental support alone, and 23% with gender-equitable attitudes alone. Together, the estimated prevalence of child abuse in the presence of all three accelerators was 11%. Compared with no accelerators, experiencing all three accelerators was associated with 64% lower prevalence of child abuse. Among males, in the absence of accelerators, the estimated prevalence of child abuse (physical or emotional) was 27%, and with gender-equitable attitudes it was 12%, which corresponds to 55% lower prevalence of child abuse. These cumulative associations from significant accelerators are depicted in Fig 1 for females and Fig 2 for males.

**Table 3:** Estimated prevalences of negative outcomes in the presence of no, one, or all accelerators among females (includes covariates and sample weights).

		Adjusted probability (%)	95% CI	Adjusted prevalence difference (%)	95% CI	Adjusted prevalence ratio	95% CI
IPV (physical or emotional)	No accelerators	16	(12, 19)	ref	–	ref	–
	Food security only	11	(8, 14)	-5	(-8, -2)	0.70	(0.54, 0.86)
	Parental support only	15	(8, 21)	-1	(-6, 4)	0.93	(0.62, 1.25)
	Gender-equitable attitudes only	13	(10, 15)	-3	(-8, 2)	0.81	(0.54, 1.07)
	All accelerators	8	(5,12)	-8	(-13, -2)	0.51	(0.23, 0.79)
Peer violence (physical or emotional)	No accelerators	28	(24, 33)	ref	–	ref	–
	Food security only	23	(18, 27)	-5	(-11, 0)	0.81	(0.62, 1.00)
	Parental support only	25	(19, 32)	-3	(-9, 3)	0.90	(0.69, 1.11)
	Gender-equitable attitudes only	21	(15, 26)	-7	(-13, -2)	0.74	(0.56, 0.92)
	All accelerators	14	(10, 19)	-14	(-22, -6)	0.49	(0.29, 0.70)
Sexual violence	No accelerators	15	(11, 18)	ref	–	ref	–
	Food security only	9	(6, 12)	-6	(-9, -2)	0.61	(0.42, 0.81)
	Parental support only	16	(8, 24)	2	(-6, 9)	1.12	(0.60, 1.64)
	Gender-equitable attitudes only	10	(7, 13)	-5	(-8, -2)	0.68	(0.50, 0.85)
	All accelerators	7	(3, 10)	-8	(-13, -3)	0.46	(0.20, 0.73)
Child abuse (physical or emotional)	No accelerators	30	(27, 34)	ref	–	ref	–
	Food security only	23	(19, 28)	-7	(-12, -2)	0.77	(0.61, 0.92)
	Parental support only	20	(14, 26)	-10	(-17, -3)	0.67	(0.46, 0.88)
	Gender-equitable attitudes only	23	(18, 28)	-7	(-12, -2)	0.77	(0.60, 0.94)
	All accelerators	11	(6, 15)	-20	(-26, -13)	0.36	(0.18, 0.53)
Inconsistent condom use	No accelerators	27	(23, 31)	ref	–	ref	–
	Food security only	21	(17, 25)	-6	(-11, -1)	0.78	(0.60, 0.95)
	Parental support only	22	(16, 28)	-5	(-9, 0)	0.83	(0.65, 1.01)
	Gender-equitable attitudes only	29	(24, 34)	2	(-2, 6)	1.08	(0.92, 1.24)
	All accelerators	19	(14, 23)	-8	(-15, -1)	0.69	(0.48, 0.91)
Age-disparate or transactional sex	No accelerators	14	(11, 18)	ref	–	ref	–
	Food security only	10	(7, 14)	-4	(-8, -1)	0.71	(0.51, 0.92)
	Parental support only	13	(9, 18)	-1	(-5, 3)	0.93	(0.68, 1.18)
	Gender-equitable attitudes only	17	(14, 20)	3	(-2, 7)	1.18	(0.83, 1.52)
	All accelerators	11	(8, 15)	-3	(-9, 3)	0.79	(0.43, 1.15)
Early sex (<16) or early pregnancy (<20)	No accelerators	12	(8, 16)	ref	–	ref	–
	Food security only	7	(3, 11)	-5	(-11, 1)	0.57	(0.16, 0.97)
	Parental support only	15	(5, 26)	3	(-7, 13)	1.26	(0.44, 2.08)
	Gender-equitable attitudes only	8	(4, 12)	-4	(-8, -1)	0.67	(0.41, 0.93)
	All accelerators	6	(2, 9)	-6	(-12, -1)	0.48	(0.12, 0.84)
Mental distress	No accelerators	48	(41, 54)	ref	–	ref	–
	Food security only	38	(32, 44)	-10	(-15, -4)	0.80	(0.69, 0.91)
	Parental support only	47	(38, 56)	-1	(-8, 7)	0.99	(0.83, 1.14)
	Gender-equitable attitudes only	39	(33, 45)	-8	(-16, -1)	0.82	(0.69, 0.96)
	All accelerators	30	(24, 36)	-18	(-28, -7)	0.63	(0.45, 0.81)
Not in school or paid work	No accelerators	23	(19, 28)	ref	–	ref	–
	Food security only	15	(11, 18)	-9	(-14, -4)	0.62	(0.46, 0.79)
	Parental support only	30	(23, 36)	6	(0, 13)	1.27	(0.97, 1.56)
	Gender-equitable attitudes only	22	(17, 27)	-1	(-6, 3)	0.94	(0.74, 1.13)
	All accelerators	18	(13,23)	-5	(-13, 3)	0.78	(0.47 1.08)
Child marriage (before age 18)	No accelerators	3	(2, 4)	ref	–	ref	–
	Food security only	1	(0, 1)	-2	(-4, -1)	0.21	(0.03, 0.39)
	Parental support only	1	(0, 2)	-2	(-4, -1)	0.29	(0.00, 0.58)
	Gender-equitable attitudes only	1	(0, 2)	-2	(-4, -1)	0.29	(0.04, 0.54)
	All accelerators	0	(0, 0)	-3	(-4, -2)	0.02	(0.00, 0.04)

CI = Confidence Interval.

**Table 4:** Estimated prevalences of negative outcomes in the presence of gender-equitable attitudes among males (includes covariates and sample weights).

		Adjusted probability (%)	95% CI	Adjusted probability difference (%)	95% CI	Adjusted probability ratio	95% CI
IPV (physical or emotional)	No accelerator	15	(10, 20)	ref		ref	–
	Gender-equitable attitudes	7	(5, 10)	-7	(-12, -3)	0.50	(0.30, 0.71)
Peer violence (physical or emotional)	No accelerator	34	(29, 39)	ref		ref	–
	Gender-equitable attitudes	17	(12, 23)	-16	(-24, -9)	0.52	(0.35, 0.68)
Sexual violence	No accelerator	9	(6, 12)	ref		ref	–
	Gender-equitable attitudes	4	(2, 6)	-5	(-9, -2)	0.42	(0.18, 0.67)
Child abuse (physical or emotional)	No accelerator	27	(22, 32)	ref		ref	–
	Gender-equitable attitudes	12	(7, 17)	-15	(-22, -7)	0.45	(0.25, 0.65)
Multiple sexual partners	No accelerator	19	(14, 23)	ref		ref	–
	Gender-equitable attitudes	10	(7, 13)	-9	(-14, -4)	0.51	(0.35, 0.68)
Inconsistent condom use	No accelerator	17	(14, 21)	ref		ref	–
	Gender-equitable attitudes	9	(6, 13)	-8	(-13, -3)	0.54	(0.32, 0.75)
Age-disparate or transactional sex	No accelerator	9	(5, 13)	ref		ref	–
	Gender-equitable attitudes	4	(3, 6)	-5	(-9, 0)	0.49	(0.22, 0.77)

CI = Confidence Interval.

### Sensitivity analyses

Sensitivity tests were conducted by running multivariable logistic regressions, including only hypothesised accelerators and covariates that were significant in either the univariate regressions or the full multivariable logistic regressions (S3 Table). These analyses aimed to assess whether the results remained consistent when using more parsimonious models, given the rarity of some study outcomes and the associated risk of overfitting. The overall findings of these analyses were the same as the main analysis.

## Discussion

### Key results

To our knowledge, this is the first study to identify potential accelerators for adolescents and young people in Namibia. Females with household food security and parental support had lower prevalences of multiple adverse outcomes. Also, both males and females with gender-equitable attitudes had lower prevalences of multiple adverse outcomes. When these protective factors are combined, there is a powerful impact in reducing the prevalences of multiple negative outcomes for adolescent girls. Combined, the identified accelerators were associated with lower prevalences of 10 out of 12 adverse outcomes among females. Among males, there were lower prevalences of 7 out of 10 adverse outcomes associated with gender-equitable attitudes (the only protective factor that was identified as an accelerator for adolescent boys and young men).

### Limitations

Principally, the cross-sectional design of this study limits the ability to establish the directionality of associations, which makes it challenging to rule out the possibility of reverse causality. To best capture temporality, we restricted the analysis to accelerators and outcomes with at least one component measured in the previous 12 months as opposed to exclusive lifetime exposures and outcomes; the exception to this was the measure for child marriage, since the prevalence was too low to calculate a past-year measure. Further, most of the relationships tested in our analyses have implausible reverse causality (e.g., inconsistent condom use affecting parental support). Where this reverse relationship is possible, claims on causality are interpreted accordingly. Additionally, longitudinal cohort studies in other countries have identified similar accelerator findings for changes in protective factors between groups [11,26–34] and within individuals [35]. These similar findings strengthen our confidence in the validity of our findings.

Missing data were overall low (less than 3%, S4 Table) but may still introduce bias by under- or over-estimating values. Previous large-scale surveys in Namibia, including the Population-based HIV Impact Assessment, have noted likely underreporting of violence, a common issue in this area of research [1,36]. Similarly, the questionnaires rely on self-reported measures, which could introduce social desirability bias or other non-response bias. To mitigate these limitations, the surveys were administered in a private location away from other household members and other household members were not told the purpose of the survey [1]. Further, male and female sampling was conducted in different primary sampling units to avoid interviewing both violence victim and perpetrator from the same community (i.e., cases of opposite-sex, interpersonal violence between similarly aged adolescents and young people) [1].

### Interpretation

Our findings suggest that when experienced in combination, the identified accelerators can have cumulative effects on the breadth (number) of outcomes and the depth of impact (additional benefit above each accelerator alone on singular outcomes) for adolescent girls and young women. Previous analysis has also suggested that these effects are optimised by implementing two or three interventions and that the gains may plateau or have diminishing returns with each additional intervention thereafter [37].

One such intervention for improving both food security and gender-equitable attitudes – the two strongest accelerators for females and the latter applying to males as well – is gender-transformative social protection [38]. Social protection aims to reduce and prevent the negative effects of poverty and can be gender transformative when programmes incorporate elements to reduce vulnerability from gender inequality [38].

One key element of social protection is social assistance, which is commonly in the form of cash, food, and in-kind transfers provided either directly to individuals or households. These forms of assistance have been shown to build resilience and reduce risk behaviours; in particular, there is evidence that receipt of cash transfers increases per capita expenditure on food [39] and cash transfers have well-documented benefits globally for food security, health, parenting, education, gender equality, and violence prevention [40–43]. There is also a growing number of studies showing how cash transfers can reduce HIV-related risk behaviours (e.g., the protective pathway of keeping girls in school) and, in a limited number of studies, HIV and HSV2 incidence in adolescent girls and young women [44,45]. Design features, such as giving cash to female household members, can facilitate improvements in gender-equitable attitudes through improved agency in relationship dynamics and purchasing power and increased spending on child health and educational needs [38]. Beyond the accelerators presented in this paper, cash transfers are frequently identified as an accelerator for adolescent health and wellbeing outcomes, including violence and HIV-related outcomes, in other African countries [11,31,33,46–51]. Relatedly, HIV-sensitive social protection is one of the key approaches identified in the UNAIDS strategy for ending inequality and HIV and this approach is especially important for adolescent girls in contexts of high HIV risk [52]. Therefore, cash transfers could have potentially large impacts on multiple outcomes for adolescents in Namibia.

Social protection, including cash transfer programmes, remains an important policy priority in Namibia, including to address food insecurity [53]. Notably, the Government of Namibia has prioritised the scale-up of a universal child grant within its national plan for social protection (2021–2030) as its first objective and strategy [54]. The child grant received 18% of the N$6.5B (~US$14.1M) expenditure on non-contributory social protection in the 2022–23 fiscal year [55]. Nonetheless, nearly 60% of children eligible to receive a child grant did not receive any support [56] and, therefore, efforts are needed to expand access to social protection, particularly for the most vulnerable households. Social protection systems can also integrate delivery of other gender-transformative interventions (such as parenting programmes) and this structure has been identified as an effective design of intervention packages among younger children [57].

Moreover, adolescents in Namibia may especially benefit from the introduction of parenting interventions to improve parental support. The World Health Organization (WHO) recently launched a set of guidelines on parenting interventions, which have consistent evidence of effectiveness in reducing child maltreatment and harsh parenting and in promoting positive parenting practices [58]. Furthermore, WHO has endorsed a set of open-access parenting interventions, Parenting for Lifelong Health [59], which is being scaled-up across the continent and other emerging economies [60]. These parenting interventions are being adapted to digital/hybrid delivery which, if effective, would provide a cost-effective approach to scale-up of the intervention and would significantly facilitate assistance for harder-to-reach populations [61].

Parenting interventions can also have components or designs dedicated to promoting positive gender norms, such as through promotion for the active involvement of fathers [62,63], or promotion of dialogue and shared decisions between caregivers [64]. Our findings suggest that the implementation of parenting interventions may lead to significant gains in multiple outcomes for girls (via gender-equitable attitudes and parental support) and boys (via gender-equitable attitudes). During the early COVID-related lockdowns, parenting interventions were shown to be quickly adaptable. Further, materials on parenting support have been promoted and distributed by the Office of the Namibian First Lady Monica Geingos [65], thus demonstrating political support for these interventions. Parenting interventions are also included within PEPFAR’s strategy for orphans and vulnerable children in Namibia to prevent HIV and sexual violence [66].

Implicit to the accelerator hypothesis is that intervening on these accelerators can be both highly impactful and cost-effective by simultaneously reducing multiple pathways of vulnerability [11]. Recent modelling has found that scaling-up parenting support through routine services in areas with high violence is likely to be cost-effective [67]. Additionally, the combined provision of parenting support with an economic strengthening component (such as cash transfers) is likely to be more cost-effective than parenting support alone [67]. This economic evidence further supports the value of implementing combination interventions, particularly cash transfers and parenting programmes, to improve multiple domains of an adolescent’s life. These data on 1) the impact of these packaged interventions on multiple outcomes and 2) their cost-effectiveness can be pivotal in gaining support from policymakers. Policy decisions often occur in contexts where policymakers are often tasked with addressing multiple demands on constrained budgets. Having these data establishes a value-for-money and facilitates the prioritisation of these interventions for implementation.

### Generalisability & conclusions

Having nationally representative data from the Violence Against Children & Youth Survey significantly strengthens the generalisability of our findings. Nonetheless, longitudinal studies and randomised trials in Namibia will be important to evaluate 1) the effectiveness of combined interventions that deliver these accelerators (e.g., cash transfers plus parenting programmes) and 2) whether these effects are sufficiently large to then impact on multiple development targets for adolescents. Research is needed to fill evidence gaps on these pathways for change (e.g., causal mediation analysis) and how combining interventions may provide added effectiveness. Other operational research is needed on scaling these interventions and optimising their design elements (e.g., digital or hybrid delivery) to ensure effectiveness with low-cost implementation.

Given the ongoing economic and global crises, innovative approaches to global development are critical to addressing the multiple vulnerabilities that adolescents face in their transition to adulthood. Our study finds that adolescents (especially girls) with access to INSPIRE provisions experience lower rates of violence, HIV risk, not being in education, and other outcomes. These findings suggest priority protective factors (food security, gender-equitable attitudes, and parental support) in improving multiple outcomes among adolescents and young people. Interventions to improve these protective factors, such as gender-transformative cash-plus programmes, can build on, and be delivered through, existing social protection infrastructures in the country. Intervening with these programmes could have substantial impacts in accelerating progress to achieve multiple SDGs for adolescents, young people, and the next generation of Namibians.

## Supporting information

S1 Table Variable Coding. Combination strategies for questionnaire items on hypothesised accelerators, outcomes, and covariates. (DOCX)

S2 Table Outcome correlations. Spearman’s correlation matrix of outcomes. (DOCX)

S3 Table Sensitivity analyses. Multivariable logistic regressions with hypothesised accelerators and covariates that were significant in either univariate regressions or in complete multivariable logistic regressions with all variables. (DOCX)

S4 Table Missing data. Prevalence of the sample with missing data for each outcome. (DOCX)
