# Supplementary material for: Accelerators to reduce violence, HIV risk, and early pregnancy among adolescents and young people in Namibia: A cross-sectional analysis of the Violence Against Children & Youth Survey
Source: PLOS Glob Public Health. 2025 May 20;5(5):e0004633. doi: 10.1371/journal.pgph.0004633 (PMC12091739; doi:10.1371/journal.pgph.0004633)
Supplement: S1 Table — (DOCX) [file pgph.0004633.s001.docx]

**S1 Table: Variable Coding.**

| **Hypothesised Accelerator** | **Question *(Household Survey (H) or Individual Survey (Q))*** | **Combination Strategy** |
| --- | --- | --- |
| **Gender-equitable attitudes** | Q42A. Do you believe only men, not women, should decide when to have sex?  Q42B. Do you believe if someone insults a boy or man, he should defend his reputation with force if he needs to?  Q42C. Do you believe there are times when a woman should be beaten?  Q42D. Do you believe women who carry condoms have sex with a lot of men?  Q42E. Do you believe a woman should tolerate violence to keep her family together?  Q43A. In your opinion, is a husband justified in hitting or beating his wife in the following situation: If she goes out without telling him?  Q43B. In your opinion, is a husband justified in hitting or beating his wife in the following situation: If she neglects the children?  Q43C. In your opinion, is a husband justified in hitting or beating his wife in the following situation: If she argues with him?  Q43D. In your opinion, is a husband justified in hitting or beating his wife in the following situation: If she refuses to have sex with him?  Q43E. In your opinion, is a husband justified in hitting or beating his wife in the following situation: If she burns the food? | A binary variable was coded for having exclusive positive gender norms if 'No' was recorded for all ten questions. |
| **Parental support** | Q22. Have you seen your biological mother in the past 12 months?  Q23. How easy or difficult is/was it to talk to your mother individually about things that really bother you? Would you say very easy, easy, difficult, very difficult, or do not have/never had a relationship with her?  Q24. How close do/did you feel to your biological mother? Would you say very close, close, not close, or do not have/never had a relationship with her?  Q32. Have you seen your biological father in the past 12 months?  Q33. How easy or difficult is/was it to talk to your father individually about things that really bother you? Would you say very easy, easy, difficult, very difficult, or do not have/never had a relationship with him?  Q34. How close do/did you feel to your biological father? Would you say very close, close, not close, or do not have/never had a relationship with him? | Each parental relationship was coded affirmatively if the child noted easy or very easy communication (Q23/33) and a close or very close relationship with this parent (Q24/34) and had seen them in the previous year (Q22/Q32). Parenting support variable was coded for having met these criteria for at least one parent. Children with one deceased parent were coded based on the relationship with the living parent. Children with both parents deceased (<3.5% of the total sample) were coded as not having parental support. Questions were not available in relation to another primary caregiver. |
| **Household food security** | Q9A. Do you think your household has enough money for food?  H52. Did you ever cut the size of the meals of child(ren) living in your household because there was not enough food or money?  H53. Did the child(ren) living in your household ever skip meals because there was not enough food or money? | Combined measure of adolescent-reported food affordability (Q9A) and household-head-reported food sufficiency (H52-H53). Food sufficiency is denoted if the answer is ‘No’ to both H52 and H53. A binary variable for household food security was coded if the household had both food affordability and sufficiency. |
|  |  |  |
| **Outcome** | **Question *(Household Survey (H) or Individual Survey (Q))*** | **Combination Strategy** |
| **Intimate partner violence (IPV) victimisation (physical or emotional)** | Q100A. Has a current or past boyfriend, romantic partner, or husband ever: Slapped, pushed, shoved, shook, or intentionally threw something at you to hurt you? A1. Has this happened in the past 12 months?  Q100B. Has a current or past boyfriend, romantic partner, or husband ever: Punched, kicked, whipped, or beat you with an object? B1. Has this happened in the past 12 months?  Q100C. Has a current or past boyfriend, romantic partner, or husband ever: Strangled, smothered, tried to drown you, or burned you intentionally? C1. Has this happened in the past 12 months?  Q100D. Has a current or past boyfriend, romantic partner, or husband ever: Used or threatened you with a knife, panga, gun or other weapon? D1. Has this happened in the past 12 months?  Q310A: Has a current or past boyfriend, romantic partner, or husband ever: insulted, humiliated, or made fun of you in front of others?  Q310B. Has a current or past boyfriend, romantic partner, or husband: Kept you from having your own money?  Q310C. Has a current or past boyfriend, romantic partner, or husband: Tried to keep you from seeing or talking to your family or friends?  Q310D. Has a current or past boyfriend, romantic partner, or husband ever: Kept track of you by demanding to know where you were and what you were doing?  Q310E. Has a current or past boyfriend, romantic partner, or husband ever: Made threats to physically harm you?  Q312. Did this [most recent time, Q110] happen in the past 12 months? | IPV victimisation was coded has having experienced physical (Q100) or emotional (Q310) violence from an intimate partner in the past 12 months. Missing values were recoded to zero to account for those never partnered. |
| **Peer violence victimisation (physical or emotional)** | Q110A. Has a person your own age ever: Slapped, pushed, shoved, shook, or intentionally threw something at you to hurt you?  Q110B. Has a person your own age ever: Punched, kicked, whipped, or beat you with an object?  Q110C. Has a person your own age ever: Strangled, smothered, tried to drown you, or burned you intentionally?  Q110D. Has a person your own age ever: Used or threated you with a knife, panga, gun, or other weapon?  Q112. Did this [most recent time, Q110] happen in the past 12 months?  Q115. Did this [first time happen] in the past 12 months?  Q315A. In the past 12 months, has someone your own age done any of these things to you: made you get scared or feel really bad because they were calling you names, saying mean things to you, or saying they didn’t want you around?  Q315B. In the past 12 months, has someone your own age done any of these things to you: told lies or spread rumors about you, or tried to make others dislike you?  Q315C. In the past 12 months, has someone your own age done any of these things to you: kept you out of things on purpose, excluded you from their group of friends, or completely ignored you? | A binary variable was created for physical or emotional violence from peers for cases occurring in the past 12 months. |
| **Sexual violence victimisation** | Q602. Now think about the last time this [touching without permission] happened. Did this happen to you within the past 12 months?  Q609. Did this [touching without permission - first time] happen to you within the past 12 months?  Q702. Now think about the last time this [attempted forced sex] happened. Did this happen to you within the past 12 months?  Q709. Did this [attempted forced sex - first time] happen to you within the past 12 months?  Q802. Now think about the last time this [physically forced sex] happened. Did this happen to you within the past 12 months?  Q810. Did this [physically forced sex - first time] happen to you within the past 12 months?  Q902. Now think about the last time this [pressured into sex] happened. Did this happen to you within the past 12 months?  Q909. Did this [pressured into sex - first time] happen to you within the past 12 months?  Q1002. Now think about the last time this [alcohol-facilitated forced sex] happened. Did this happen to you within the past 12 months?  Q1009. Did this [alcohol-facilitated forced sex - first time] happen to you within the past 12 months? | A binary variable was created for experiencing any form of sexual violence in the past 12 months. |
| **Child abuse (physical or emotional)** | Q46A. In the past 12 months, has a parent or adult caregiver punished or corrected you by shouting, yelling, or screaming at you; calling you offensive names, such as ‘dumb’ or ‘lazy’; taking away food; or ignoring you for several hours?  Q46B. In the past 12 months, has a parent or adult caregiver punished or corrected you by shaking, hitting, spanking, or slapping you anywhere on your body with a bare hand or a hard object?  Q120A. Has a parent, adult caregiver, or other adult relative ever: slapped, pushed, shoved, shook, pulled hair, twisted arm, pinched, or intentionally threw something at you to hurt you?  Q120B. Has a parent, adult caregiver, or other adult relative ever: punched, kicked, whipped, or beat you with an object?  Q120C. Has a parent, adult caregiver, or other adult relative ever: strangled, smothered, tried to drown you, or burned you intentionally?  Q120D. Has a parent, adult caregiver, or other adult relative ever: used or threatened you with a knife, panga, gun or other weapon?  Q122. Did this [most recent time] happen in the past 12 months?  Q126. Did this [first time] happen in the past 12 months?  Q300A. Has a parent, adult caregiver or other adult relative ever: told you that you were not loved, or did not deserve to be loved?  Q300B. Has a parent, adult caregiver or other adult relative ever: said they wished you had never been born or were dead?  Q300C. Has a parent, adult caregiver or other adult relative ever: ridiculed you or put you down, for example said that you were stupid or useless?  Q302. Did this [most recent time] happen in the past 12 months?  Q306. Did this [first time] happen in the past 12 months? | A binary variable was created for physical or emotional child abuse if the caregiver had used violent discipline (Q46) or perpetrated physical (Q120) or emotional violence (Q300) toward the child in the past 12 months. |
| **Multiple sexual partners** | Q425. How many people have you had sex within the past 12 months? | A binary variable was created for having no or one sexual partner, or 2+ sexual partners in the previous year. |
| **Inconsistent condom use** | Q412. Have you ever had sex?  Q429. In the past 12 months when you had sex with this person (most recent sexual partner), how often did you use a condom? Would you say always, sometimes, or never?  Q436. In the past 12 months when you had sex with this second person (second most recent sexual partner), how often did you use a condom? Would you say always, sometimes, or never?  Q443. In the past 12 months when you had sex with this third person (third most recent sexual partner), how often did you use a condom? Would you say always, sometimes, or never? | A respondent is noted as having inconsistent condom use in the past 12 months if condoms were not always used with the three most recent sexual partners in the past year. |
| **Age-disparate or transactional sex** | Q433. In the past 12 months, did you ever have sex with this person [most recent sexual partner] mainly in order to get things that you need such as money, gifts, or other things that are important to you?  Q440. In the past 12 months, did you ever have sex with this person [second most recent sexual partner] mainly in order to get things that you need such as money, gifts, or other things that are important to you?  Q447. In the past 12 months, did you ever have sex with this person [third most recent sexual partner] mainly in order to get things that you need such as money, gifts, or other things that are important to you?  Q507. In the past 12 months, how many times did you have sex with someone mainly in order to get things that you need such as money, gifts, or other things that are important to you?  Q427. Think about the most recent person who you had sex with in the past 12 months. How old was this person? Please give your best guess.  Q435. Now think back to a second person with whom you had sex in the past 12 months. How old was this second person? Please give your best guess.  Q442. Now think back to a third person with whom you had sex in the past 12 months. How old was this third person? Please give your best guess. | A binary measure for transactional sex was created as answering affirmatively to Q443, Q440, or Q447 or as a value of 1 or more to Q507. A binary variable for age-disparate sex was coded as affirmative if there is a five or more year age-gap between the respondent and any of their three most recent sexual partners in the past 12 months. A combined variable was created for having had either age-disparate or transactional sex in the past 12 months. |
| **Early sexual debut (before age 16) or early pregnancy (before age 20)** | Q413. How old were you when you had sex for the very first time?  Q419. How old were you the first time that you got pregnant? | Among the sample <16 years old, early sex in the previous year was calculated by subtracting current age from age at first sex. Early sex in the past year was coded affirmatively if the difference was within one year. Among the sample <20 years old, adolescent pregnancy in the previous year was calculated from subtracting current age (Q2) from age at first pregnancy. Adolescent pregnancy in the past year was coded affirmatively if the difference was within one year. A total variable was created either had early sexual debut or early pregnancy in the past year. |
| **Heavy drinking** | Q1401. In the past 30 days, on how many days did you have 4 or more drinks of alcohol in a row? | Heavy drinking was coded affirmatively if the respondent answered with one or more days in the past 30 days. |
| **Mental distress** | Q1405A. During the past 30 days, how often did you feel NERVOUS: all the time, most of the time, some of the time, a little of the time, or none of the time?  Q1405B. During the past 30 days, how often did you feel HOPELESS: all the time, most of the time, some of the time, a little of the time, or none of the time?  Q1405C. During the past 30 days, how often did you feel RESTLESS: all the time, most of the time, some of the time, a little of the time, or none of the time?  1405D. During the past 30 days, how often did you feel SO SAD THAT NOTHING COULD CHEER YOU UP: all the time, most of the time, some of the time, a little of the time, or none of the time?  Q1405E. During the past 30 days, how often did you feel THAT EVERYTHING WAS AN EFFORT: all the time, most of the time, some of the time, a little of the time, or none of the time?  Q1405F. During the past 30 days, how often did you feel WORTHLESS: all the time, most of the time, some of the time, a little of the time, or none of the time? | This variable coded mental distress using Kessler-6 Psychological Distress Scale (Prochaska et al., 2012). Scores to each question were recoded based on a scale from 0 (none of the time) to 4 (all the time). Moderate or severe mental distress was coded affirmatively if respondents scored between 5-24. A binary variable was created for no mental distress (scoring 0-4) or having mental distress (scoring 5-24) in the past 30 days.  *Prochaska, J. J., Sung, H. Y., Max, W., Shi, Y., & Ong, M. (2012). Validity study of the K6 scale as a measure of moderate mental distress based on mental health treatment need and utilization. International journal of methods in psychiatric research, 21(2), 88–97. https://doi.org/10.1002/mpr.1349* |
| **Not in school or paid work** | Q4. Are you currently attending school?  Q11. At any time during the past 12 months did you engage in any work as an employee, or self-employed individual?  Q13. Did you receive money for this work? | A binary variable was coded to reflect if the respondent was neither currently attending school nor involved in paid work. |
| **Child marriage** | Q36. Have you ever been married or lived with someone as if you were married?  Q37. How old were you when you first got married or started living together? | A binary variable was coded affirmatively if the respondent had ever been married since before age 18. |
|  |  |  |
| **Covariates** | **Question *(Household Survey (H) or Individual Survey (Q))*** | **Combination Strategy** |
| **Age group** | Q2. How old were you at your last birthday? | Age group was created for those 18-24 years (reference age 13-17). |
| **Orphanhood** | Q15. Is your biological mother still alive?  Q25. Is your biological father still alive? | Respondents were coded as orphaned if one or both biological parents were deceased. |
| **DREAMS-priority district** | [Used pre-coded strata variable and encode numeric values] | Priority regions were oversampled in survey, including those living in Khomas, Oshikoto, or Zambezi. |
| **Urban** | [Used pre-coded strata variable and encode numeric values] | Rural living was used as the reference value. |
| **Household poverty** | H8. What type of fuel does your household mainly use for cooking?  H6. Do you share this [toilet] facility with other households?  H4. What is the main source of drinking water for members of your household?  H7A. Does your household have electricity?  H9. Record the main material of the dwelling floor.  H10. Record the main material of the roof.  H11. Record the main material of the walls.  H7B-H7G. Does your household have a radio, TV, phone (mobile or landline), refrigerator?  H14. Does any member of your household own: bicycle, motorcycle/motorbike, animal-drawn cart or oxcart?  H14G. Does any member of your household own a car/truck? | Principal component analysis was used for the following components of multidimensional poverty:  Clean/improved cooking fuel (Including electricity, liquefied petroleum gas, or biogas.)  Private sanitation  Safe drinking water (Including Piped (tap) water into dwelling; piped (tap) water on site or in yard/plot; public tap/standpipe; private tube well/borehole; protected dug well; protected spring and bottled water)  Electricity  Improved housing (Deprived if floor is made of mud/clay/earth, sand, or dung; Deprived if dwelling has no roof or walls or if either the roof or walls are constructed using natural materials such as cane, palm/trunks, sod/mud, dirt, grass/reeds, thatch, bamboo, sticks, or rudimentary materials such as carton, plastic/ polythene sheeting, bamboo with mud/stone with mud, loosely packed stones, adobe not covered, raw/reused wood, plywood, cardboard, unburnt brick, or canvas/tent.)  Asset ownership (Deprived if own zero or one items in the list. Households were also coded as not having asset deprivation if they owned a car or truck.)  Wealth index scores were assigned to each household and based on the range were grouped into quintiles. The lowest two quintiles were noted as those living in poverty, which represented the bottom 40% of the sample.  *OPHI (2018). Global Multidimensional Poverty Index 2018: The Most Detailed Picture to Date of the World’s Poorest People. Report. Oxford Poverty and Human Development Initiative (OPHI), University of Oxford. ISBN 978-1-912291-12-0.*  *Namibia Statistical Agency. (2021). Namibia Multidimensional Poverty Index (MPI) Report 2021. Available at: https://ophi.org.uk/wp-content/uploads/Namibia_MPI_report_2021.pdf* |
| **Female-headed household** | H2. Interviewer record the sex of the head of household. | Male-headed household was used as the reference value. |
