# Supplementary material for: Accelerators to reduce violence, HIV risk, and early pregnancy among adolescents and young people in Namibia: A cross-sectional analysis of the Violence Against Children & Youth Survey
Source: PLOS Glob Public Health. 2025 May 20;5(5):e0004633. doi: 10.1371/journal.pgph.0004633 (PMC12091739; doi:10.1371/journal.pgph.0004633)
Supplement: S2 Table — (DOCX) [file pgph.0004633.s002.docx]

**S2 Table: Correlations between outcomes.**

|  | (A) | (B) | (C) | (D) | (E) | (F) |
| --- | --- | --- | --- | --- | --- | --- |
| (A) Intimate partner violence | 1 |  |  |  |  |  |
| (B) Peer violence | 0.0557* | 1 |  |  |  |  |
| (C) Sexual violence | 0.2008* | 0.1979* | 1 |  |  |  |
| (D) Child abuse | 0.1099* | **0.3689*** | 0.1893* | 1 |  |  |
| (E) Multiple sexual partners | 0.1304* | -0.0186 | 0.1009* | 0.0055 | 1 |  |
| (F) Inconsistent condom use | 0.1963* | -0.1656* | 0.0339* | -0.0956* | 0.1691* | 1 |
| (G) Age-disparate or transactional sex | 0.1372* | -0.1205* | 0.0109 | -0.0834* | 0.0700* | **0.4033*** |
| (H) Early sex or early pregnancy | 0.1001* | -0.0469* | 0.0380* | -0.0146 | 0.0363 | **0.4093*** |
| (I) Heavy drinking | 0.1373* | -0.0289* | 0.0775* | -0.0111 | 0.1779* | 0.1003* |
| (J) Mental distress | 0.1363* | 0.0390* | 0.1196* | 0.0973* | 0.0403* | 0.0984* |
| (K) Not in school or paid work | 0.0519* | -0.1513* | 0.007 | -0.1227* | 0.0498* | 0.2749* |
| (L) Child marriage | 0.0800* | -0.0531* | -0.0041 | -0.0198 | 0.0144 | 0.1664* |
|  |  |  |  |  |  |  |
|  | (G) | (H) | (I) | (J) | (K) | (L) |
| (A) Intimate partner violence |  |  |  |  |  |  |
| (B) Peer violence |  |  |  |  |  |  |
| (C) Sexual violence |  |  |  |  |  |  |
| (D) Child abuse |  |  |  |  |  |  |
| (E) Multiple sexual partners |  |  |  |  |  |  |
| (F) Inconsistent condom use, don't know HIV status |  |  |  |  |  |  |
| (G) Age-disparate or transactional sex | 1 |  |  |  |  |  |
| (H) Early sex or early pregnancy | 0.2585* | 1 |  |  |  |  |
| (I) Heavy drinking | 0.0690* | -0.0086 | 1 |  |  |  |
| (J) Mental distress | 0.0750* | 0.0266 | 0.0801* | 1 |  |  |
| (K) Not in school or paid work | 0.2130* | 0.2309* | 0.0485* | 0.0803* | 1 |  |
| (L) Child marriage | 0.1211* | 0.1457* | -0.024 | 0.0071 | 0.1215* | 1 |
| * p<0.05, bolded associations \|r\|>0.30 | | | | |  |  |
| Only three outcome pairs (peer violence victimisation and child abuse as well as inconsistent condom use correlated to age-disparate or transactional sex and early sexual debut or early pregnancy) have a correlation \|r\|>0.30. The correlation for the latter two pairs is due to the combination strategies for the measures, where the same value is applied for the 57% of the weighted sample that was not sexually active (either abstinent in the past year or over lifetime). | | | | | | |
