# Supplementary material for: Accelerators to reduce violence, HIV risk, and early pregnancy among adolescents and young people in Namibia: A cross-sectional analysis of the Violence Against Children & Youth Survey
Source: PLOS Glob Public Health. 2025 May 20;5(5):e0004633. doi: 10.1371/journal.pgph.0004633 (PMC12091739; doi:10.1371/journal.pgph.0004633)
Supplement: S3 Table — (DOCX) [file pgph.0004633.s003.docx]

**S3 Table: Sensitivity analyses.**

|  | Females | | | Males | | |
| --- | --- | --- | --- | --- | --- | --- |
|  | AOR | (95% CI) | p-value | AOR | (95% CI) | p-value |
| **IPV (physical or emotional; n=4134F, 964M)** |  |  |  |  |  |  |
| Household food security | 0.64 | (0.48, 0.85) | 0.002 | - | - | - |
| Parental support | - | - | - | - | - | - |
| Gender-equitable attitudes | - | - | - | 0.43 | (0.26, 0.73) | 0.003 |
| **Peer violence (physical or emotional; n=4134F, 964M)** |  |  |  |  |  |  |
| Household food security | - | - | - | 0.55 | (0.33, 0.93) | 0.027 |
| Parental support | - | - | - | - | - | - |
| Gender-equitable attitudes | 0.56 | (0.38, 0.83) | 0.004 | 0.39 | (0.24, 0.64) | <0.001 |
| **Sexual violence (n=4134F, 964M)** |  |  |  |  |  |  |
| Household food security | 0.59 | (0.42, 0.82) | 0.002 | - | - | - |
| Parental support | - | - | - | 0.16 | (0.27 1.26) | 0.162 |
| Gender-equitable attitudes | 0.63 | (0.47, 0.84) | 0.002 | 0.40 | (0.20, 0.78) | 0.009 |
| **Child abuse (physical or emotional; n=4134F, 964M)** |  |  |  |  |  |  |
| Household food security | 0.69 | (0.52, 0.91) | 0.009 | - | - | - |
| Parental support | 0.55 | (0.35, 0.87) | 0.010 | - | - | - |
| Gender-equitable attitudes | 0.68 | (0.50, 0.92) | 0.014 | 0.33 | (0.18, 0.61) | 0.001 |
| **Multiple sexual partners (n=4067F, 937M)** |  |  |  |  |  |  |
| Household food security | - | - | - | - | - | - |
| Parental support | - | - | - | - | - | - |
| Gender-equitable attitudes | - | - | - | 0.44 | (0.29, 0.67) | <0.001 |
| **Inconsistent condom use (n=4057F, 933M)** |  |  |  |  |  |  |
| Household food security | 0.64 | (0.47, 0.87) | 0.005 | - | - | - |
| Parental support | - | - | - | - | - | - |
| Gender-equitable attitudes | - | - | - | 0.49 | (0.30, 0.80) | 0.007 |
| **Age-disparate or transactional sex (n=4134F, 964M)** |  |  |  |  |  |  |
| Household food security | 0.55 | (0.41, 0.76) | <0.001 | - | - | - |
| Parental support | - | - | - | - | - | - |
| Gender-equitable attitudes | - | - | - | 0.47 | (0.24, 0.91) | 0.028 |
| **Early sex (<16) or early pregnancy (<20) (n=2481F, -)** |  |  |  |  |  |  |
| Household food security | 0.52 | (0.24, 1.13) | 0.099 | - | - | - |
| Parental support | - | - | - | - | - | - |
| Gender-equitable attitudes | 0.59 | (0.38, 0.92) | 0.02 | - | - | - |
| **Binge drinking (n=4034F, 956M)** |  |  |  |  |  |  |
| Household food security | - | - | - | - | - | - |
| Parental support | - | - | - | - | - | - |
| Gender-equitable attitudes | - | - | - | - | - | - |
| **Mental distress (n=4126F, 964M)** |  |  |  |  |  |  |
| Household food security | 0.64 | (0.49, 0.82) | 0.001 | - | - | - |
| Parental support | - | - | - | - | - | - |
| Gender-equitable attitudes | 0.68 | (0.48, 0.94) | 0.022 | - | - | - |
| **Not in school or paid work (n=4064F, 958M)** |  |  |  |  |  |  |
| Household food security | 0.52 | (0.37, 0.74) | <0.001 | - | - | - |
| Parental support | - | - | - | - | - | - |
| Gender-equitable attitudes | - | - | - | - | - | - |
| **Child marriage (before age 18; n=4107F, -)** |  |  |  |  |  |  |
| Household food security | 0.20 | (0.08, 0.47) | <0.001 | - | - | - |
| Parental support | 0.28 | (0.11, 0.75) | 0.012 | - | - | - |
| Gender-equitable attitudes | 0.28 | (0.11, 0.71) | 0.008 | - | - | - |
| AOR = Adjusted Odds Ratio; CI = Confidence Interval; IPV = Intimate Partner Violence; F = Female Sample; M = Male Sample | | | | | | |
