# Supplementary material for: Accelerators to reduce violence, HIV risk, and early pregnancy among adolescents and young people in Namibia: A cross-sectional analysis of the Violence Against Children & Youth Survey
Source: PLOS Glob Public Health. 2025 May 20;5(5):e0004633. doi: 10.1371/journal.pgph.0004633 (PMC12091739; doi:10.1371/journal.pgph.0004633)
Supplement: S4 Table — (DOCX) [file pgph.0004633.s004.docx]

**S4 Table: Missing data by variable.**

| **Variable** | **Missing (%)** |
| --- | --- |
| **Hypothesised Accelerators** |  |
| Household food security | 0.0 |
| Parental support | 0.0 |
| Gender-equitable attitudes | 1.2 |
|  |  |
| **Outcomes** |  |
| IPV victimisation (physical or emotional, 12mon) | 0.0 |
| Peer violence victimisation (physical or emotional, 12mon) | 0.0 |
| Sexual violence victimisation (12mon) | 0.0 |
| Child abuse (physical or emotional, 12mon) | 0.0 |
| Multiple sexual partners (12mon) | 2.0 |
| Inconsistent condom use (12mon) | 2.3 |
| Age-disparate or transactional sex (12mon) | 0.0 |
| Early sex (<16) or early pregnancy (<20) (12mon) | 0.0 |
| Binge drinking (30 days) | 2.1 |
| Moderate or severe mental health distress (30 days) | 0.2 |
| Not in school or paid work (12mon) | 1.6 |
| Child marriage (before age 18, lifetime) | 1.4 |
|  |  |
| **Sociodemographic Covariates** |  |
| Age 13-17 (Binary) | 0.0 |
| Orphanhood | 0.1 |
| Urban | 0.0 |
| Household poverty (lowest two quintiles) | 0.0 |
| Oversampling in priority district | 0.0 |
| Female-headed household | 0.0 |
